# Impact of Increased SGLT2 Inhibitor Uptake in CKD Management in Denmark: Modeling National Patient Benefits and Cost Savings

**DOI:** 10.1016/j.xkme.2026.101363

**Published:** 2026-04-15

**Authors:** Renée Hangaard Olesen, Nicholas Carlson, Jens Søndergaard, Ellen Linnea Freese Ballegaard, Rikke Borg, Lars Holger Ehlers

**Affiliations:** 1 Nordic Institute of Health Economics, Aarhus, Denmark; 2 Department of Nephrology and Endocrinology, Copenhagen University Hospital, Copenhagen, Denmark; 3 Research Unit of General Practice, Department of Public Health, University of Southern Denmark, Odense, Denmark; 4 Department of Medicine, Zealand University Hospital, Roskilde, Denmark; 5 Department of Clinical Medicine, University of Copenhagen, Copenhagen, Denmark

**Keywords:** Chronic kidney disease, costs and cost analysis, disease management, renal replacement therapy, sodium/glucose cotransporter 2 inhibitors

## Abstract

**Rationale & Objective:**

Chronic kidney disease (CKD) poses an increasing global burden for patients and health care providers, driven by aging populations and the rising prevalence of chronic diseases. Despite strong evidence supporting benefits of sodium/glucose cotransporter 2 inhibitors (SGLT2is), uptake remains limited. The objective of this study was to model potential benefits of increased SGLT2i uptake on clinical and economic outcomes of CKD in Denmark.

**Study Design:**

A dynamic decision-analytic modeling study with a 10-year time horizon.

**Setting & Participants:**

Danish incident and prevalent populations with CKD stages G3-G5 eligible for SGLT2is.

**Exposure:**

SGLT2i treatment with uptake rates of 15%, 50%, and 90%. Assumptions about SGLT2i treatment effects were based on the EMPA-KIDNEY trial.

**Outcomes:**

The model evaluated benefit on all-cause mortality, quality-adjusted life years, cardiovascular disease, and kidney failure (KF). The broader health care impact was estimated through costs and net monetary benefit.

**Analytical Approach:**

A validated Markov state microsimulation model (CKD progression model) using 18 health states, defined by eGFR and urinary albumin-creatinine ratio thresholds defined by Kidney Disease: Improving Global Outcomes (KDIGO).

**Results:**

Based on prevalence and incidence of CKD, the model predicts a 30% increase in the population of individuals with CKD stages G3-G5 from 251,946 in 2026 to 328,414 in 2035 with the current practice. SGLT2i uptake of 15%, 50%, and 90% are associated with a 10-year risks of KF of 8.5%, 6.6%, and 4.5%, respectively, and 10-year mortality rate of 54.3%, 53.9%, and 53.5%. This results in a net monetary benefit of 204 and 438 million €, respectively, compared with uptake of 15%.

**Limitations:**

The efficacy of SGLT2is was derived from a single randomized controlled trial.

**Conclusions:**

Based on a validated CKD progression model, increased SGLT2i uptake in Denmark is associated with increased patient survival and reduced progression to KF, leading to a net benefit on health care spending within a 6-year time horizon.

## Plain-language Summary

Chronic kidney disease is becoming more common, and many patients do not receive treatments that could slow the worsening of their condition. This motivated us to explore what might happen if more people in Denmark were treated with sodium/glucose cotransporter 2 inhibitors, a therapy shown to benefit individuals with kidney disease. Using a validated model based on real patient data, we estimated how increased use of sodium/glucose cotransporter 2 inhibitors could affect health outcomes over the coming years. The analysis suggests that wider use could help people live longer, delay serious complications, and reduce pressure on the health care system. Our findings highlight that improving access to proven treatments has the potential to benefit both patients and society as kidney disease continues to grow.

The impact of chronic kidney disease (CKD) on health care is substantial and growing, driven by the increase in aging populations and the high prevalence of chronic conditions.^1^, ^2^, ^3^ By 2040, CKD may rank as the fifth leading cause of years of life lost worldwide.^4^ CKD is associated with an increased risk of kidney failure (KF), cardiovascular disease (CVD), and premature mortality.^5^, ^6^, ^7^ with costs escalating as CKD progresses.^2^^,^^8^

Sodium/glucose cotransporter 2 inhibitors (SGLT2is) have been shown to slow CKD progression and reduce the need for kidney replacement therapy (KRT). Benefits have been demonstrated in both the EMPA-KIDNEY and DAPA-CKD trials in CKD populations regardless of diabetes status.^5^^,^^9^^,^^10^ These results strongly support SGLT2is as a crucial therapy in CKD management across a broad spectrum of patients with CKD.

Despite strong evidence supporting the cost-effectiveness of SGLT2is^11^, ^12^, ^13^, ^14^ and treatment advocated in current international and Danish clinical guidelines,^15^^,^^16^ uptake in Denmark is currently estimated to approximately 15%.^17^ Consequently, implementation remains suboptimal, with possible implications for long-term economic and clinical outcomes.^17^^,^^18^ Using a validated CKD progression model (CKD-PM), this study estimates the predicted impact of increased SGLT2i uptake on kidney and mortality outcomes, including the broader impact on costs to the Danish public health care system.

## Methods

A validated CKD-PM^19^ was used to estimate the prevalence of CKD in Denmark from 2026 to 2036. Prevalence rates of CKD were analyzed based on varying uptake of SGLT2is in eligible patients, with uptake ranging from current estimates of 15%^18^ to a near total implementation rate of 90%. Associated patient outcomes were estimated based on modeled rates of mortality, quality-adjusted life-years (QALYs), KF, and cardiovascular outcome events. Based on results, the estimated attributable costs and net monetary benefit (NMB) were computed for assessment of the broader impact of treatment on the Danish public health care system.

### Eligible Population

The prevalent population was defined as the diagnosed cases of CKD stages G3-G5 (based on the Kidney Disease: Improving Global Outcomes [KDIGO] categories based on estimated glomerular filtration rate [eGFR]^15^^,^^19^), and the incident population referred to the annual number patients newly diagnosed with CKD stages G3-G5. The prevalent population was based on the Danish CKD prevalence (4.62%) multiplied with the total number of people aged >18 years in Denmark, using population data from 2024.^20^, ^21^, ^22^ The incident population was estimated to have an annual mean of 27,816 based on published and unpublished data.^21^^,^^23^ The prevalent and incident populations only accounted for those who were eligible for treatment according to current guidelines.^16^ The size of the eligible population was estimated at 26.75% of the CKD population.^24^ The eligible population was adjusted at the beginning of each year to account for mortality from the previous year.

### The CKD-PM

The CKD-PM is a validated Markov state microsimulation model using 18 health states, defined by eGFR and urinary albumin-creatinine ratio thresholds based on the KDIGO categories.^15^^,^^19^ Transitions between health states are determined by projections of annual changes in eGFR and urinary albumin-creatinine ratio in the model. The model simulates CKD progression by integrating disease markers (eGFR and urinary albumin-creatinine ratio) and risk factors such as age, body mass index, glycated hemoglobin, serum cholesterol, systolic blood pressure, and comorbid conditions such as diabetes and hypertension. Additionally, it accounts for the annual risk of events by incorporating the evolution of CKD risk factors over time, estimating the likelihood of CKD-related complications and comorbid conditions, and modeling how these complications influence further disease progression.^19^ A health economic module was incorporated for assigning costs to treatments, health states, and clinical events.

The validated model was used to simulate the incident and prevalent populations with CKD separately. The baseline characteristics for the prevalent and incident CKD G3-G5 population were incorporated into the model, based on the best available evidence in a Danish context (all references are listed in Table 1 and Tables S1-S2). The base case analysis was conducted with a 10-year time horizon, reflecting SGLT2i implementation rates of 15%, 50%, and 90%. Results from the CKD progression model were proportionally weighted to reflect the burden of CKD, depending on the degree of SGLT2i uptake.

**Table 1:** Baseline Characteristics, Risk/Probabilities, and Costs

	Prevalent CKD G3-G5	Incident CKD G3-G5	Source
**Eligible CKD patients in Denmark**	59,955	7,440^a^	Danmarks Statistik^20^ (2024); Kampmann^21^ (2023); Carlson^22^ (2021); Vestergaard^23^ (2024)
**Baseline characteristics**			
Mean age, y	76	74	Kampmann^21^ (2023); Vestergaard^25^ (2021); Fraser^26^ (2015)
Male	45%	44%	Kampmann^21^ (2023); Carlson^22^ (2021); Vestergaard^25^ (2021); Fraser^26^ (2015)
Mean eGFR, mL/min/1.73 m^2^	56	54	Vestergaard^25^ (2021)
Mean UACR, mg/g	75	21	Freese Ballegaard^27^ (2024), unpublished^b^
Mean HbA1c, %	5.9	5.9	Stevens^15^ (2024), unpublished^b^
Mean BMI, kg/m^2^	28.5	28.5	Freese Ballegaard^27^ (2024)
Mean total cholesterol, mg/dL	174	174	Freese Ballegaard^27^ (2024)
Mean systolic blood pressure, mm Hg	132	132	Freese Ballegaard^27^ (2024)
CKD stage			Kampmann^21^ (2023); Carlson^22^ (2021); Vestergaard^25^ (2021); Fraser ^26^ (2015)
CKD3a	67.9%	75.8%	
CKD3b	23.8%	18.7%	
CKD4	6.6%	4.8%	
CKD5^c^	1.7%	0.7%	
Comorbid conditions			
Diabetes	19.2%	13.8%	Kampmann^21^ (2023); Carlson^22^ (2021); Vestergaard^25^ (2021)
Cardiovascular disease	26.7%	23.5%	Kampmann^21^ (2023); Carlson^22^ (2021); Vestergaard^25^ (2021)
Hypertension	47.6%	49.4%	Carlson^22^ (2021)
Congestive heart failure	10.1%	8.5%	Carlson^22^ (2021); Vestergaard^25^ (2021)
**Risk/probabilities**			
CKD progression model	CKD-PM	CKD-PM	Ramos^19^ (2024)
**Costs (annually)**			
CKD stage 3a	€1,242-2,098	€1,242-2,098	Pollock^6^ (2022)
CKD stage 3b	€1,456-2,327	€1,456-2,327	Pollock^6^ (2022)
CKD stage 4	€1,859-3,062	€1,859-3,062	Pollock^6^ (2022)
CKD-stage 5	€2,509-5,572	€2,509-5,572	Pollock^6^ (2022)
Hemodialysis	€63,455	€63,455	Danish Health Authority^28^ (2023)
Peritoneal dialysis	€36,869-42,572	€36,869-42,572	Danish Health Authority^28^ (2023)
Kidney transplantation	€37,481-44,603	€37,481-44,603	Danish Health Authority^28^ (2023)
Immunosuppressive therapy	€10,678	€10,678	Danish Health Authority^28^ (2023)
SGLT2i	€567	€567	Danish Medicines Agency^29^ (2025)
SoC	€13	€13	Danish Medicines Agency^29^ (2025)

*Note:* All baseline characteristics, costs and utilities applied in the CKD progression model are shown in Supplementary, Table S1, S2 and S6-S9. Abbreviations: CKD, chronic kidney disease; eGFR, estimated glomerular filtration rate; SGLT2i, sodium/glucose cotransporter 2 inhibitor; SoC, standard of care; UACR, urinary albumin-creatinine ratio.

a Annual number of incident patients.

b Unpublished data, Ballegaard et al (2025).

c SGLT2i effects were not applied to CKD5 (Table S4).

### Treatment Effects and Outcomes

The CKD-PM incorporates outcome events based on established prediction algorithms, risk equations, and incidence rates to estimate the annual probability of clinical events.^19^ Outcome events in the model included all-cause death, KF (including KRT), acute kidney injury, CVD (new-onset heart failure, CVD hospitalizations, and major adverse cardiac events, defined as stroke, myocardial infarction, and cardiovascular death), anemia, mineral and bone disorder, diabetes, hypertension, infections, and other adverse events. A description of all events is provided in Table S3. Patient transitions between KRT modalities and current number of patients in KRT were based on data from the Danish Registry on Regular Dialysis and Transplantation.^30^ Mortality estimates in the model were derived from all-cause mortality rates as a function of eGFR and albuminuria,^31^ as this approach best reflects observed mortality in Danish patients with CKD.^25^ In the model, treatment effects, adverse events, and treatment discontinuation of SGLT2is were derived from the EMPA-KIDNEY trial^9^ (Table S4-S5). Upon treatment discontinuation, natural disease progression was assumed, with no residual treatment effect.

### Costs

Relevant costs were applied to each of the 18 health states, clinical events, and drug acquisition related to SGLT2i and standard therapy (Table 1). The costs applied in this study were based on the principle of best available evidence, allowing a mix of data sources including Danish Diagnosis Related Group tariffs and official pharmacy purchase prices excluding value added tax.^32^^,^^33^ For CVD complications, an acute cost was applied in the year the complication occurred, followed by follow-up costs in subsequent years as long as the patient remained alive. For other complications, costs were applied annually, per event, or in the first year of occurrence, as appropriate (Table S6-S8). All costs were estimated in Danish Krones, converted to euros at an exchange rate of 7.46, and adjusted to the price year 2024. In line with current guidance for budget impact analyses, no discount was incorporated in the analysis.^33^ NMB was calculated as the willingness-to-pay threshold per QALY (λ) multiplied by the incremental QALYs, minus the incremental costs: NMB = (λ × ΔQALY) – ΔCost. This method converts the health gains into a monetary value, allowing direct comparison with additional costs. A positive NMB indicates that the intervention is considered cost-effective at the specified willingness-to-pay threshold. The NMB was assessed with a willingness-to-pay threshold of €24,178/QALY, aligned with the National Institute of Health and Care Excellence’s threshold of £20,000.^34^

### QALY

QALY is a metric used in health economics to quantify the value of health outcomes by integrating both the duration and quality of life into a single index. The quality component is represented by utility values ranging from 0 (equivalent to death) to 1 (perfect health). Utilities were applied for the specific health state for CKD stages G3-G5, dialysis, and kidney transplantation.^35^^,^^36^ These health state utilities were age-adjusted according to age-specific utilities from the general Danish population.^37^ Disutilities were incorporated once during the cycle of occurrence of each specific complication (Table S9).

### Sensitivity Analyses

Sensitivity analyses in which one or more parameters were changed simultaneously were conducted to assess the robustness of the model. These included variations in the number of incident patients within the range (17,600-40,000) observed in the data^21^^,^^23^ and adjustments based a mortality rate of 57% after 10 years, as is observed in the Danish CKD population,^25^ as well as increasing the number of eligible patients by 25%. Finally, results were evaluated under the assumptions of an 80% treatment effect and an 80% reduction in the cost of SGLT2is.

## Results

**Figure 1:**
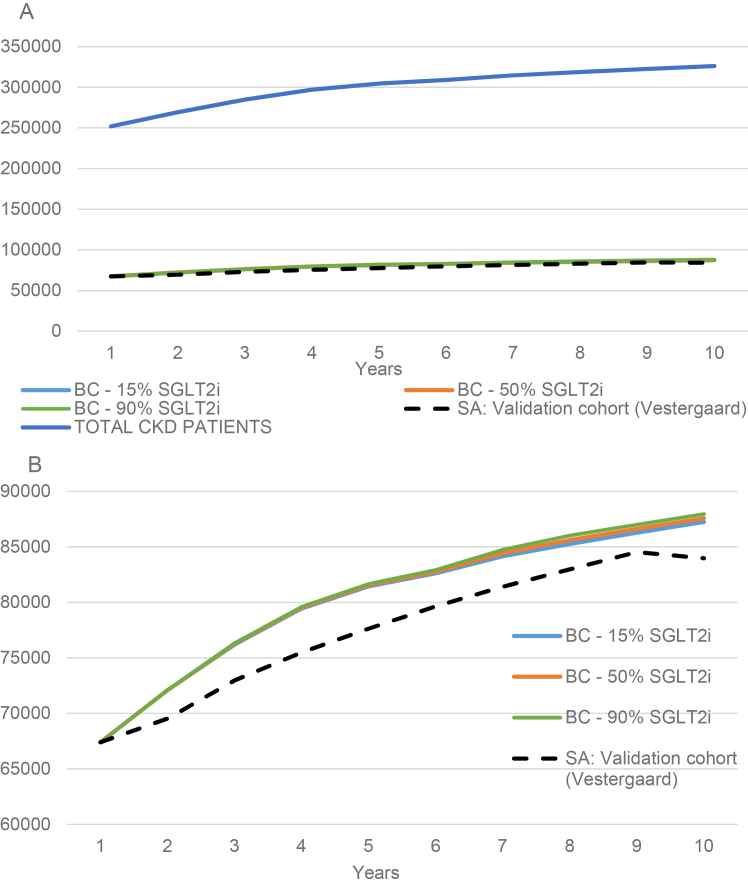
Base case estimated number of total CKD patients and eligible CKD patients. Panel A shows the total CKD patients and Panel B shows the predicted number of eligible CKD patients. The dashed line represents the estimated number of patients based on the mortality rates observed in Vestergaard et al. (2021).^25^ Abbreviations: BC, base case; CKD, chronic kidney disease; SA, scenario analysis; SGLT2i, sodium/glucose cotransporter 2 inhibitors.

Based on the validated model, the total number of patients with CKD stages G3-G5 was demonstrated to increase over the 10-year period from 251,946 in 2026 to 328,414 in 2036 (Fig 1^27^), with an increase in the estimated number of SGLT2i-eligible patients from 67,395 in 2026 to 87,256 in 2036 based on current SGLT2i uptake. This corresponds to an overall 30% increase in the total CKD G3-G5 population, and a consequent 29% increase in the number of patients eligible for SGLT2is (Table 2). Sensitivity analyses showing estimated populations dependent on incidence rates of CKD G3-G5 are provided in Table S10.

**Table 2:** Ten-Year Development in Eligible Patients and Accumulated Number of Kidney Failures, Cardiovascular events, QALYs, and Costs

SGLT2i uptake (%)	2026			2031			2036		
	15	50	90	15	50	90	15	50	90
**Eligible patients with CKD**									
	67,395	67,395	67,395	81,458	81,560	81,677	87,256	87,588	87,967
**CVD**									
MACE	801	801	801	1,979	1,982	1,985	3,380	3,406	3,435
New onset HF	337	337	337	2,220	2,202	2,182	3,180	3,133	3,078
CVD hospitalization	2,259	2,235	2,208	12,011	11,796	11,550	17,689	17,366	16,991
**KF**									
KF + KRT	382	382	382	973	888	791	2,334	1,884	1,364
Hemodialysis	375	375	375	832	747	649	1,865	1,472	1,017
Peritoneal dialysis	0	0	0	215	215	215	702	580	438
Kidney transplantation	7	7	7	373	376	379	752	660	554
**Costs** (million €)									
KRT	25.6	25.6	25.6	117.2	109.3	100.3	493.7	385.0	261.1
CVD complication	178.3	177.1	175.8	1,134.2	1,120.6	1,105.1	2,696.1	2,661.2	2,621.4
Total	542.7	554.8	568.6	3,269.5	3,255.1	3,273.1	7,815.2	7,681.1	7,528.3
**Cost-effectiveness**									
QALY gain	0	7	15	0	534	1,145	0	2,920	6,279
NMB (million €)	0	−11.9	−25.5	0	−2.8	−5.9	0	204	438

Abbreviations: CKD, chronic kidney disease; CVD, cardiovascular disease; HF, heart failure; KF, kidney failure; KRT, kidney replacement therapy; MACE, major adverse cardiac event; NMB, net monetary benefit; QALY, quality-adjusted life-year; SGLT2i, sodium/glucose cotransporter 2 inhibitor.

Based on the model, increased SGLT2i uptake rates were associated with improved survival rates, with 50% and 90% SGLT2i uptake associated with 332 and 711 more patients alive at 10 years, respectively, compared with 15% SGLT2i (Table 2), corresponding to 10-year mortality rates of 54.3%, 53.9%, and 53.5% for 15%, 50%, and 90% SGLT2i uptake respectively.

### Cardiovascular Outcomes

The modeled impact of SGLT2i uptake on cardiovascular outcomes was divergent. SGLT2i uptake of 50% and 90% were associated with an overall increase of 26 and 55 major adverse cardiac events within the 10-year period, respectively. However, a benefit of increased SGLT2i uptake was demonstrated for 10-year rates of new-onset heart failure and CVD hospitalization, with SGLT2i uptake of 50% and 90% associated with a decrease of 47 and 102 patients with new-onset heart failure, and 323 and 698 CVD hospitalizations, respectively. A summary of results is shown in Table 2.

### KF Outcomes

**Figure 2:**
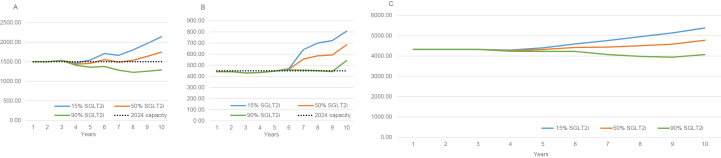
Base case estimated number of eligible patients with chronic kidney disease progressing to KF and initiating KRT. The number of patients as shown in table 2 has been adjusted based on the current prevalence of patients in KRT. It is assumed that 70% of those currently in KRT would have been eligible for SGLT2i treatment prior to initiating KRT. Panel A shows hemodialysis, Panel B shows peritoneal dialysis, and Panel C shows KRT. Abbreviations: BC, base case; HD, hemodialysis; KF; kidney failure, KRT, kidney replacement therapy; PD, peritoneal dialysis; SGLT2i, sodium/glucose cotransporter 2 inhibitor.

Based on the model and under the assumption of static SGLT2i uptake of 15%, prevalence of KRT would exceed current capacity of approximately 2500-2700 KRT patients within 5 years (Fig 2).

Overall, increase in SGLT2i uptake in eligible patients with CKD G3-G5 was associated with lower rates of KF and consequently lower rates of KRT initiation, with 10-year risks of KF of 8.5%, 6.6%, and 4.5% for SGLT2i uptake of 15%, 50%, and 90%, respectively. Comparably, SGLT2i uptakes of 50% and 90% were associated with a decrease of 607 and 1,310 patients initiating KRT, compared with SGLT2i uptake of 15% (Table 2).

### Costs, QALYs, and NMB

**Figure 3:**
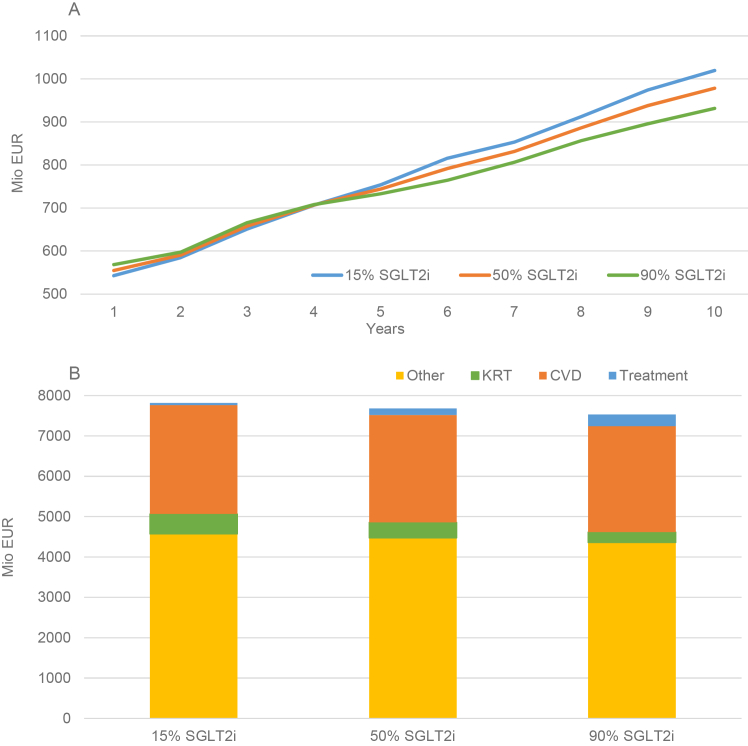
Base case annual and accumulated incremental cost of eligible patients with chronic kidney disease. Panel A shows the annual costs and Panel B shows the accumulated costs over 10 years. “Other” refers to costs related to bone and mineral disorders, infections, acute kidney injury, anemia, and adverse events (leg/foot/toe amputation). Abbreviations: CVD, cardiovascular disease; KRT, kidney replacement therapy; Mio EUR, million euros; SGLT2i, sodium/glucose cotransporter 2 inhibitor.

In the base case analysis, annual costs grew in line with the increasing number of patients and the progression of eligible individuals to KF requiring KRT. Annual costs were lowest for the 15% SGLT2i implementation until year 4, after which the 90% SGLT2i implementation became the least costly strategy (Fig 3).

Increased SGLT2i uptake was associated with reduced accumulated costs related to KRT, CVD complications, and in total over 10 years (Fig 3). Cost benefits related to CVD complications were demonstrated within 1 year, while cost benefits related to KRT were not demonstrated until the third year (data not shown), and total savings was not demonstrated until 6 years (Table 2 and Fig S1). NMB remained principally unchanged in sensitivity analyses based on a reduced SGLT2i treatment effect and in models adjusted for increased mortality rates (Table S10). Cumulative savings of increased SGLT2i uptake over 10 years in the base case amounted to €108.7-232.6 million for KRT, €34.7-74.7 million for CVD complications, and €134.1-286.9 million in total (Table 2). Reducing SGLT2i costs to 20% resulted in overall cost savings from the second year (Figure S2).

Increased uptakes of SGLT2is were also associated with a greater QALY gain (Table 2). Incorporating the QALY gain, SGLT2i uptakes of 50% and 90% were associated with cost savings from year 6 (Fig S1). Additionally, in sensitivity analyses assuming reductions in SGLT2i costs of 20%, increased uptakes of SGLT2is were associated with cost savings from the second year (Fig S2).

## Discussion

Based on a validated CKD progression model, our results demonstrate a projected increase in patients with CKD G3-G5 overall in Denmark over the next 10 years, with concurrent increase in patients eligible for SGLT2is. Based on model estimations using a population-based microsimulation approach incorporating SGLT2i treatment effects from the EMPA-KIDNEY trial,^9^ increased SGLT2i uptake was demonstrated to be associated with benefit on mortality and lower rates of CKD progression, consequently with lower numbers of patients progressing to KF and KRT. Furthermore, demonstrable annual cost savings, particularly in relation to KRT and CVD complications, were evident from 5 years.

This study has some limitations. First, there is considerable uncertainty regarding both the overall incidence of CKD G3-G5 and the number of SGLT2i-eligible CKD patients. Patient eligibility for SGLT2is based on albuminuria levels was unfortunately not included. An important limitation is the lack of prevalence estimates regarding albuminuria in the Danish CKD population. Accordingly, we report sensitivity analyses to address both uncertainties related to mortality and the overall number of eligible patients. Second, the treatment effects are estimated based on outcomes from the EMPA-KIDNEY trial, which had a relatively short active treatment and follow-up period. As such, our results depend on a single trial and may not fully capture long-term effects. Third, the precision of the CKD-PM for evaluation of SGLT2i effects is highest within 2-year time horizon of the source clinical trials. The treatment impact on the risk of death, CVD, and kidney outcomes including transitions to and from each health state and rate of events is fully mediated via the assumed treatment effect on eGFR, urinary albumin-creatinine ratio, and, in the case of CVD endpoints, assumed treatment-related changes in systolic blood pressure and hemoglobin A_1c_. Furthermore, the model outcome estimations are derived in part from source data prior to implementation of SGLT2is in CKD populations, as such attributable treatment effects do not account for possible period effects. Fourth, the CKD-PM assumes constant kidney transplantation probabilities. Thus, it projects fewer transplants when fewer patients reach KF under broader SGLT2i uptake. Because transplant capacity is likely fixed, the model may overestimate dialysis needs by not accounting for higher transplant rates among a smaller eligible population. Fifth, the CKD-PM model only aims to estimate incremental costs between treatments, and consequently only reports on cost differences between SGLT2i uptake categories. It is known that uptake of evidence-based interventions in clinical practice remains slow, with adoption of novel interventions previously reported to exceed 15 years.^38^ Instant adoption of guideline recommendations is rare, with average uptake in eligible patients reportedly 10%-20% within the first year increasing to approximately 50% by 5 years conditional on the interplay of numerous rate-defining factors.^39^ As such, uptake of SGLT2is in eligible patients beyond 50% may remain elusive irrespective of evidence of benefit. Finally, the population was restricted to CKD stages G3-G5, as reliable demographics and characteristics remain limited for patients with lesser CKD stages. Consequently, the findings may not be generalizable to patients with CKD stages G1-G2, for whom comprehensive data are needed to ensure valid conclusions. However, a key strength is that the reported results are modeled on comprehensive and detailed data on patients with CKD stages G3-G5.

Our study estimates a rise in eligible CKD patients over the next 10 years. Previous microsimulation studies have similarly predicted a global rise in CKD prevalence overall, albeit with predicted decline in CKD G3-G5 reported for Denmark between 2022 and 2027.^4^ Population projections based on demographics in Denmark forecast substantial growth in population age, particularly in populations aged >80 years.^20^ However, management of CKD is currently evolving, with introductions of numerous novel kidney-protective medications with plausible benefit on kidney outcomes.^15^ As such, observed discrepancies only highlight overt uncertainties related to predictions on future prevalence of CKD in Denmark, with important implications for projections of eligible patient numbers.

Our results align closely with the clinical and budgetary impact of treatment with SGLT2is observed in previous studies. In a French study evaluating the budget impact of implementing SGLT2is for all patients with elevated albuminuria, the findings demonstrated accumulated budget savings, fewer patients progressing to KF, reduced hospitalizations related to heart failure, and fewer deaths over a 5-year period.^40^ Similarly, an Australian study demonstrated an association of increased uptake of SGLT2is with slower CKD progression and lower mortality due to CVD and kidney disease.^41^ Costs associated with SGLT2is have also been shown to be offset by benefits of delaying CKD progression in multiple cohorts.^11^^,^^14^^,^^40^^,^^42^ Overall, our results are consistent with prior observations supporting a beneficial association of SGLT2i uptake with disease progression and mortality rates and simultaneously benefit on health care costs through moderating effects on the need for costly treatments including KRT. However, our economic results cannot directly be compared to classic cost-effectiveness studies of SGLT2is in CKD as these are typically static cohort projections, whereas this is dynamic modeling that estimates the expected future growth in the incident CKD population. As such, the modeled savings due to postponing KRT are higher.

Modeling the benefits of increased SGLT2i uptake has significant implications for patients, but also a significant impact on CKD health-related costs. Therefore the current limited use of SGLT2is and substantial proportion of undiagnosed and/or unrecognized CKD is an area that needs attention and further research,^43^ especially in the primary care sector, where most CKD patients are managed. Clinical trials have demonstrated the importance of early identification and treatment in individuals at high-risk in primary care for prevention of CKD progression, with specific implications for reducing the burden of specialized care.^9^^,^^10^^,^^44^ As our results are limited to CKD stages G3-G5, the broader population, including those eligible for SGLT2is in CKD stages G1-G2, may place an even greater demand on primary care and other non-nephrology specialties. This highlights the need for further research and enhanced strategies supportive of early diagnosis and treatment.

Overall, our results highlight the potential of SGLT2is to improve clinical outcomes while reducing health care resource utilization, such as hospitalizations and the progression to KF. As health care systems face increasing pressure, the hope is that guideline-recommended treatment with SGLT2is will play a key role in addressing future resource demands. Future research on prevalence and incidence rates of CKD across all stages is needed to estimate clinical outcomes and costs for the broader, general CKD population more precisely. Additionally, studies exploring the short- and long-term impact of SGLT2is in real-world settings will provide future insight.

In conclusion, based on population estimates of CKD G3-G5 in Denmark, our results model the benefit of increased SGLT2i uptake on associated patient outcomes leading to a reduction in health care costs within a 5- to 6-year horizon. Consequently, our results highlight potential value of a broader uptake of SGLT2is for management of a growing population of patients with CKD in Denmark.
