# Supplementary material for: Impact of Increased SGLT2 Inhibitor Uptake in CKD Management in Denmark: Modeling National Patient Benefits and Cost Savings
Source: Kidney Med. 2026 Apr 15;8(6):101363. doi: 10.1016/j.xkme.2026.101363 (PMC13218231; doi:10.1016/j.xkme.2026.101363)
Supplement: Supplementary File (PDF) — Table S1-S10. Figure S1-S2. [file mmc1.pdf]

Supplementary Material

Title: Impact of Increased SGLT2-Inhihitor Uptake in CKD Management in Denmark: Modelling National Patient Benefits and Cost Savings

Authors: Renée Hangaard Olesen, Nicholas Carlson, Jens Søndergaard, Ellen Linnea Freese Ballegaard, Rikke Borg, Lars Holger Ehlers

Table S1. Baseline characteristics for the prevalent CKD population

Table S2. Baseline characteristics for the incident CKD population

Table S3. Outcome events applied in the model

Table S4. Projected changes in eGFR and uACR values over 1 year in patients receiving SGLT2i as add on to standard of care and standard of care alone.

Table S5. Incremental effects of SGLT2 and standard of care on other risk factors across health states.

Table S6. Health states and medicine costs

Table S7. Kidney failure cost and probability

Table S8. Clinical event costs

Table S9. Utility and disutility

Table S10. Sensitivity analyses

Figure S1. Accumulated incremental costs and net monetary benefit with the current price of SGLT2i

Figure S2. Accumulated incremental costs and net monetary benefit with an 80% price reduction of SGLT2i

**Table S1.** Baseline characteristics for the prevalent CKD population		
	**Input**	**Source**
**Demographic variables**		
Mean age	76.4	(1)
Gender (male)	45.25	(1,2)
Race<br>- Caucasians<br>- Black<br>- Asians/Indians<br>- Hispanic-Caribbeans	 92.70%<br>1.33%<br>5.53%<br>0.44%	(3)
**Clinical risk factors**		
Smoking	19.0%	(4)
Mean eGFR (ml/min per 1.73 m2)	56	(5)
Median uACR (mg/g)	75	(4)
Mean HbA1c (%-point)	5.9	(6)
Mean BMI	28.5	(4)
TC (mg/dL)	174	(4)
HDL (mg/dL)	58	(4)
SBP (mmHg)	132.3	(4)
Height (m)	173.5	(7)
Controlled hypertension	140	(4)
HbA1c threshold for DM (%-point)	6,5	(8)
**eGFR class distribution**		
3a-eGFR G3a (%)	67.9%	(1,2,5)
3b-eGFR G3b (%)	23.8%	
4-eGFR G4 (%)	6.6%	
5-eGFR G5 (%)	1.7%	
**uACR class distribution**		
1-uACR A1	72%	(1,5)
2-uACR A2	22%	
3-uACR A3	6%	
**History of comorbidities**		
Diabetes (%)	19.2%	(1,2)
CV disease	26.7%	(1,2)
Hypertension	47.6%	(2)
Congestive heart failure	10.1%	(5)
**Clinical management (medication only)**		
Treatment for hypertension	80.2%	(4,6)
Statins	50.5%	(4,6)
**Non-diabetic**		
Pre-diabetes (%)	28.8%	(9)

**Table S2.** Baseline characteristics for the incident CKD population		
	**Input**	**Source**
**Demographic variables**		
Median age	73.95	(1,5,10)
Gender (male) (%)	43.90	(1,5,11)
Race (%)<br>- Caucasians<br>- Black<br>- Asians/Indians<br>- Hispanic-Caribbeans	 92.70%<br>1.33%<br>5.53%<br>0.44%	(3)
**Clinical risk factors**		
Smoking (%)	19.0%	(4)
Mean eGFR (ml/min per 1.73 m2)	54	(5)
Mean uACR (mg/g)	21	*
Mean HbA1c (%-point)	5.9	*
Mean BMI	28.5	(4)
Mean TC (mg/dL	174	(4)
Mean HDL (mg/dL)	58	(4)
Mean SBP (mmHg)	132	(4)
Mean Height (m)	173.5	(7)
Controlled hypertension (mmHg)	140	(4)
HbA1c threshold for DM (%-point)	6.5	(8)
**eGFR class distribution**		
3a-eGFR G3a (%)	75.8	(1,5,11)
3b-eGFR G3b (%)	18.7	
4-eGFR G4 (%)	4.8	
5-eGFR G5 (%)	0.7	
**uACR class distribution**		
1-uACR A1(%)	60%	*(5)
2-uACR A2 (%)	30%	
3-uACR A3 (%)	10%	
**History of comorbidities**		
Diabetes (%)	13.8%	(1,5)
CV disease (%)	23.5%	(1,5)
Hypertension (%)	49.4%	(2)
Congestive heart failure (%)	8.5%	(2,5)
**Clinical management (medication only)**		
Treatment for hypertension (%)	72.5%	*
Statins (%)	46%	*
**Non-diabetic**		
Pre-diabetes (%)	20,7%	(9)
*Unpublished data (Ballegaard et al 2025)		

Outcome events applied in the model

The clinical events in the model were based on literature review as described in the validation of the CKD-progression model (12).

**Table S3.** Outcome events applied in the model	
**Outcome**	**Description**
Mortality	All-cause mortality based on eGFR and uACR (13).
Kidney failure (Kidney replacement therapy)	Reaching an eGFR of 15 mL/min per 1.73 m2, as per the KDIGO classification. To determine whether patients would undergo KRT, a 6-variable risk equation for KRT was applied (14).
Cardiovascular disease	The risk of CV outcomes in the model was estimated using the 'CKD Patch' engines (15). The outcomes included acute MI, TIA stroke, unstable angina, heart failure and peripheral artery disease. Furthermore, MACE was included defined as stroke, myocardial infarction and cardiovascular death.
Comorbidities	Diabetes (Q-diabetes model) (16) and hypertension (17).
Mineral and bone disorders	Fractures (18), secondary hyperthyroidism (19), hypocalcemia (20) and hyperphosphatemia (19).
Infections	Respiratory, urogenital, gastro-intestinal and bloodstream infections (21).
Acute kidney injury	Defined by diagnostic code (22).
Anemia	Defined as recorded blood Hb level <12 g/dl in women and <13 g/dl in men (23).
Other adverse events	Hyperuricemia/gout, hyperkaliemia and metabolic acidosis (24).

EMPA-KIDNEY inputs applied in the model

The EMPA-KIDNEY inputs in the model are described as in a previous health economic evaluation of SGLT2 inhibitors using the CKD-PM (12).

Patients' disease progression through the KDIGO health states was modelled according to projected treatment-specific changes in eGFR and uACR values, while alive and on treatment. These were derived from the observed total annual eGFR slopes and changes at 18 months in uACR from the EMPA-KIDNEY trial as shown in Table S4.

Furthermore, changes in other risk factors including HbA1c, bodyweight, body mass index and systolic blood pressure that impact the occurrence of complications and death were programmed into the model. These were derived from the EMPA-KIDNEY trial as shown in Table S5.

Treatment effects of SGLT2i on acute kidney injury (hazard ratio 0.78; 95% confidence interval (CI) 0.60–1.00 vs standard of care alone) and hospitalization for heart failure (hazard ratio 0.80; 95% CI 0.60–1.06 vs standard of care alone) were derived from the EMPA-KIDNEY trial.

Rates of lower limb amputation and discontinuation of treatment from the EMPA-KIDNEY trial were included in the model. Lower limb amputation rates were 0.43 and 0.29 per 100 patient-years with SGLT2i and standard of care, respectively. Annual discontinuation rates of treatment were 12.56 and 14.16 per 100 patient-years with SGLT2i and standard of care, respectively.

Table S4. Projected changes in eGFR and uACR values over 1 year in patients receiving SGLT2i as add on to standard of care and standard of care alone.

KDIGO	SGLT2 10 mg once daily plus standard of care			Placebo plus standard of care		
	**A1**	**A2**	**A3**	**A1**	**A2**	**A3**
**Mean (95% CI) change in eGFR (mL/min/1.73 m^2)**						
**G2**	NA	-2.2 (-3.23, -1.14)	-3.39 (-3.06, -2.81)	NA	-2.76 (-3.92, -1.59)	-5.14 (-5.7, -4.58)
**G3a**	NA	-1.60 (-2.32, -0.89)	-3.45 (-3.91, -2.98)	NA	-2.29 (-3.04, -1.55)	-4.66 (-5.14, - 4.19)
**G3b**	-0.58 (-0.96, -0.19)	-1.04 (-1.40, -0.67)	-2.90 (--3.20, -2.60)	-0.83 (-1.2 - 0.46)	-1.56 (-1.92, -1.20)	-4.11 (-4.42, -3.80)
**G4**	-0.32 (-0.87, 0.22)	-0,62 (-1.04, -0.19)	-2.76 (-3.08, -2.45)	-0.15 (-0.71, 0,40)	-0.85 (-1.27, -0.43)	-3.76 (-4.09, -3.44)
**Mean (95% CI) change in uACR**						
**G2**	NA	1.26 (0.93, 1.70)	0.67 (0.56, 0.79)	NA	0.93 (0.67, 1.29)	0.75 (0.63, 0.89)
**G3a**	NA	0.87 (0.71, 1.06)	0.53 (0.46, 0.61)	NA	0.99 (0.80, 1.23)	0.71 (0.62, 0.81)
**G3b**	1.62 (1.44, 1.82)	0.84 (0.76, 0.94)	0.62 (0.56, 0.67)	1.65 (1.47, 1.85)	1.09 (0.98, 1.22)	0.81 (0.74, 0.88)
**G4**	2.08 (1.76, 2.44)	1.03 (0.91, 1.18)	0.68 (0.62, 0.74)	2.44 (2.07, 2.87)	1.51 (1.33, 1.71)	0.95 (0.86, 1.04)
Abbreviations: CI, confidence interval; eGFR, estimated glomerular filtration rate; NA, not						

available, uACR, urine albumin-to-creatinine ratio.

**Table S5**. Incremental effects of SGLT2 and standard of care on other risk factors across health states.		
**Risk factors**	**SGLT2 10 mg once daily plus standard of care**	**Placebo plus standard of care**
Glycated hemoglobin, mmol/mol	-0.5585 (-0.08252, -0.2917)	-0.1455 (-0.4142, 0.1232)
Weight, kg	-1.5525 (-1.7375, -1.3676)	-0.679 (-0.8648, -0.4933)
Body mass index (calculated), kg/m^3	-0.55	-0.24
Systolic blood pressure, mmHg	-3.915 (-4.3245, -3.5056)	-1.2913 (-1.17019, -0.8808)

All annual KDIGO stage costs are based on Pollock et al. 2022 (26), where resource use for outpatient visits, emergency department visits, and general practitioner visits are included in the annual costs. Hospital admissions and acute treatments are not included, as they are considered part of the complications in the model. The following sources were used for the cost estimates:

- Outpatient visits (2,344 DKK) – Average cost for an outpatient visit in Denmark (26).
- Emergency department visits – assumed to have the same cost as an outpatient visit.
- General practitioner visits (160 DKK) – assumed to be a consultation (27).

Table S6. Health states and medicine costs

KDIGO-stage	Type of cost	Input (EUR 2024)	Source
G3a*A1	Annual	1,242	(25–27)
G3a*A2	Annual	1,168	(25–27)
G3a*A3	Annual	2,098	(25–27)
G3b*A1	Annual	1,456	(25–27)
G3b*A2	Annual	1,707	(25–27)
G3b*A3	Annual	2,327	(25–27)
G4*A1	Annual	1,859	(25–27)
G4*A2	Annual	1,987	(25–27)
G4*A3	Annual	3,062	(25–27)
G5*A1	Annual	2,509	(25–27)
G5*A2	Annual	2,366	(25–27)
G5*A3	Annual	5,572	(25–27)
**Treatment cost**			
SGLT2i (Empagliflozin)	Annual	567	(28)
Standard therapy (RAAS-treatment)	Annual	13	(28)

**Table S7.** Kidney failure cost and probability			
	Type of cost	Input (EUR)	Source
Conservative therapy (medicine)	Annual	757	(28)
Continuous ambulatory peritoneal dialysis (CAPD)	Annual	36,869	(29)
% patients on CAPD	-	9%	(30)
Automated peritoneal dialysis (APD)	Annual	42,572	(29)
% patients on APD	-	10%	(30)
Haemodialysis	Annual	63,455	DRG 11PR10 (31)
Kidney transplant (living donor)	Event	44,603	DRG: 11MP02, 11MP01, 11MP10 (30,31)
Kidney transplant (deceased donor)	Event	37,481	DRG: 11MP02, 11MP01, 11MA10 (30,31)
% of patients getting living donor renal transplant	-	30%	(30)
Acute kidney injury (AKI) - outpatient	Event	208	DRG 11MA98 (31)
Acute kidney injury (AKI) - hospitalization	Event	6,609	DRG 11MA01 (31)
Immunosuppressive Therapy for KT	Annual	10,678	DRG 16MP04 (31)
Peritonitis	Event	4,608	DRG 06MA10 (31)
AV access Thrombosis	Event	3,383	DRG 05MA12 (31)
Bloodstream infections	Event	6,180	DRG 18MA08 (31)

**Table S8.** Clinical event costs			
	Type of cost	Input (EUR)	Source
Myocardial infarction	Acute	22,727	(32)
Myocardial infarction (+1 year)	Follow-up	554	(32)
Unstable angina	Acute	554	(32)
Unstable angina (+1 year)	Follow-up	554	(32)
Stroke	Acute	18,826	(32)
Stroke (+ 1 year)	Follow-up	3.345	(32)
Congestive heart failure (CHF) (hospitalizations)	Acute	19,711	(32)
Congestive heart failure (CHF) (hospitalizations) (+ 1 year)	Follow-up	517	(32)
Transient Ischemic Attack (TIA)	Acute	3,193	DRG 01MA13 (31)
PAD	Acute	2,605	(33)
PAD (+ 1 year)	Follow-up	2.042	(33)
Metabolic acidosis	Annual	1598	(34)
Hyperkalaemia	Event	3,366	DRG 10MA04 (31)
Hyperphosphatemia	Annual	315	(35)
Secondary Hyperparathyroidism	Annual	1,142	(35)
Hyperuricemia/Gout	Annual	2,726	(36)
Hypocalcaemia	Annual	315	(35)
Hip fractures	Event	10,924	DRG 08MP28 (31)
Other fractures	Event	5,595	DRG 08MA02 (31)
Respiratory infections	Event	162	(37)
Urinary tract infection	Event	52	(38)
Skin and soft tissue infections	Event	1,867	(39)
Gastrointestinal infection	Event	198	(40)
Muscular infections	Event	5,836	DRG 08MA13 (31)
Nervous system	Event	9,772	DRG 01MA03 (31)
Sepsis	Event	6,743	DRG 18MA01 (31)
Anaemia (chronic)	Annual	4,724	(27,28,41)
**Adverse events**			
Leg/foot amputation	Event	15,850	DRG 05MP17 (31)
Toe amputation	Event	6,664	DRG 05MP18 (31)

**Table S9.** Utility and disutility		
	**Input**	**Source**
**Utility**		
CKD-stages<br>- CKD 3<br>- CKD 4<br>- CKD 5	 0.80<br>0.74<br>0.73	 (42)
Kidney failure<br>- Peritoneal dialysis<br>- Hemodialysis<br>- Kidney transplant	 0.58<br>0.56<br>0.71	 (38,43)
**Disutility**		
Myocardial infarction	-0.0550	(44,45)
Unstable angina	-0.0900	(44,45)
Stroke	-0.1640	(44,45)
Heart failure (hospitalization)	-0.1080	(44,45)
TIA	-0.0700	(46)
PAD and PVD	-0.0610	(45,47)
Hip fractures and other fractures	-0.0680	(46)
Anemia	-0.0800	(48)
Acute kidney injury	-0.0380	(46)
Immunosuppressive therapy	-0.0100	(49)
Leg/toe/foot amputation	-0.1172	(49)

**Table S10.** Sensitivity analyses									
	**2026**			**2031**			**2036**		
**SGLT2i uptake (%)**	**15**	**50**	**90**	**15**	**50**	**90**	**15**	**50**	**90**
**Eligible patients with CKD**									
Validation cohort (Vestergaard)*	67395	-	-	77642	-	-	83977	-	-
Incident (Max range)	70655	70655	70655	96323	96433	96559	112418	112822	113284
Incident (Min range)	64663	64663	64663	68993	69089	69198	66157	66429	66740
33% eligible for SGLT2i	84244	84244	84244	101822	101950	102096	109070	109484	109959
**Costs** (Mio. €)									
Validation cohort (Vestergaard)	542.7	554.8	568.6	3113.2	3127.0	3142.8	7559.5	7410.4	7242.8
Total 80% SGLT2i effect	542.7	554.8	568.6	3239.5	3255.1	3273.1	7815.2	7681.1	7528.3
**Cost-effectiveness**									
NMB 20% SGLT2i cost (Mio. €)	0	-1.6	-3.4	0	45.2	96.9	0	292.5	626.8
Abbreviations: SGLT2i; Sodium-glucose cotransporter-2 inhibitors, CKD; Chronic kidney disease, NMB; Net monetary benefit. *Same number of eligible patients regardless of implementation of SGLT2i as the same mortality was applied for SGLT2 and standard of care.									

Figure S1. Accumulated incremental costs and net monetary benefit with the current price of SGLT2i

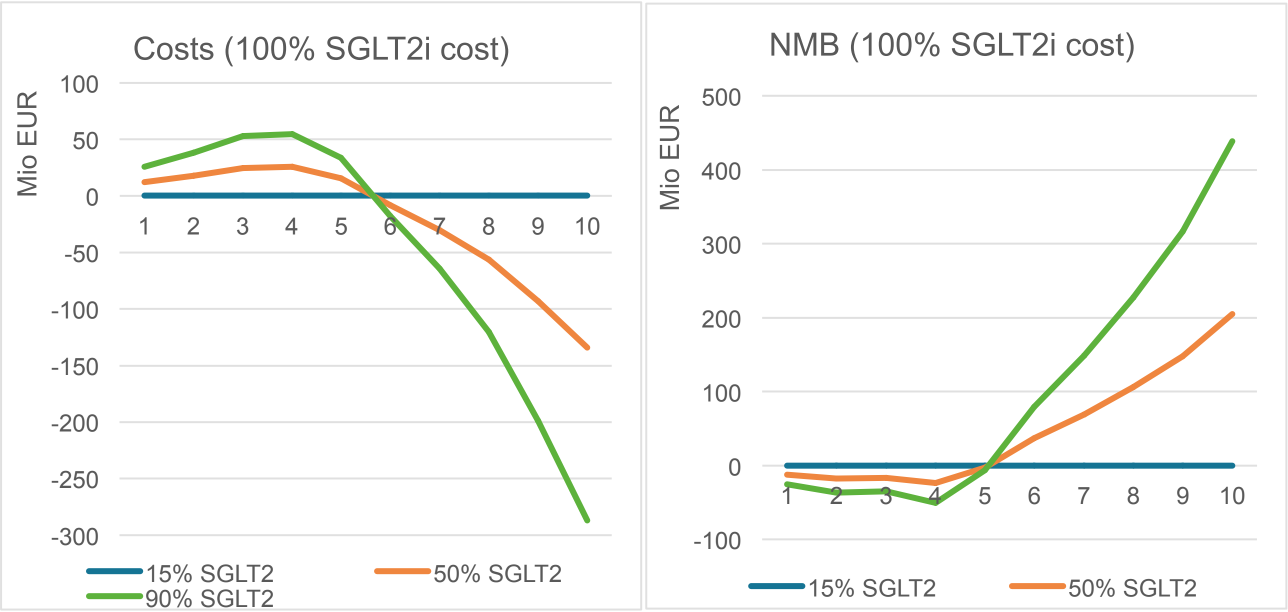

Figure S2. Accumulated incremental costs and net monetary benefit with an 80% price reduction of SGLT2i

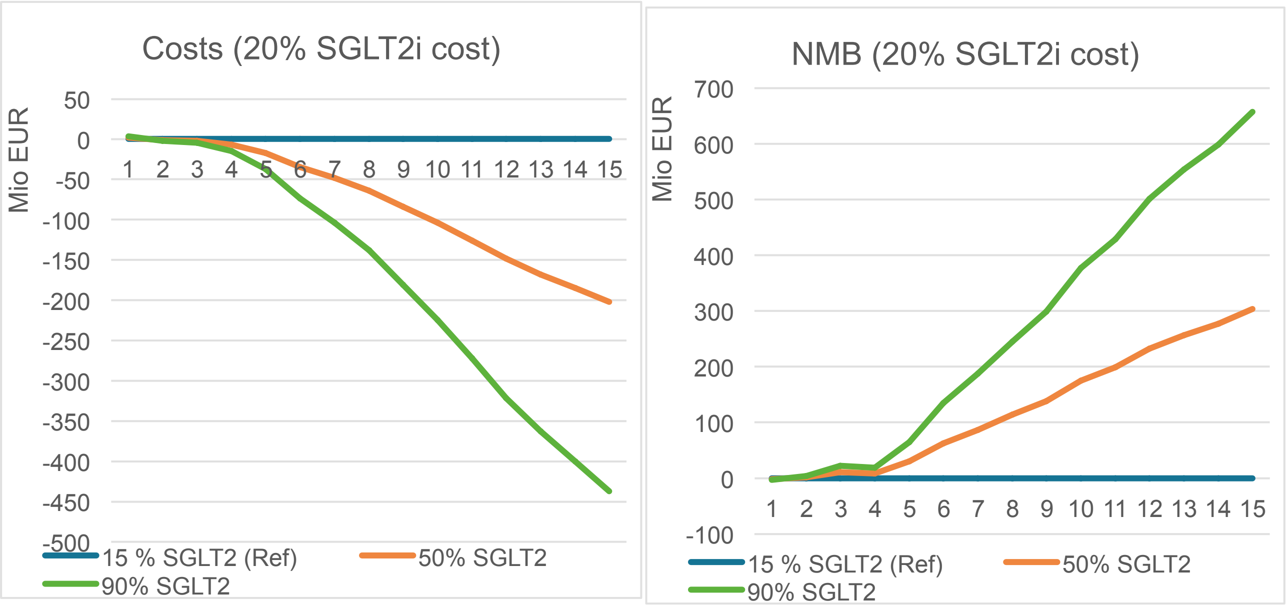

Supplementary References

1. Kampmann JD, Heaf JG, Mogensen CB, Mickley H, Wolff DL, Brandt F. Prevalence and incidence of chronic kidney disease stage 3–5 – results from KidDiCo. BMC Nephrol. 19. januar 2023;24(1):17.

2. Carlson N, Nelveg□Kristensen K □E., Freese Ballegaard E, Feldt□Rasmussen B, Hornum M, Kamper A □Lise, m.fl. Increased vulnerability to COVID□19 in chronic kidney disease. J Intern Med. juli 2021;290(1):166–78.

3. Danmarks Statistik [Internet]. 2025 [henvist 21. februar 2025]. Befolkningen 1. januar efter tid, Hovedpersonens fødeland og Hovedpersonen herkomst. Tilgængelig hos: https://www.statistikbanken.dk/KRYDS3

4. Freese Ballegaard EL, Carlson N, Buus Jørgensen M, Sørensen IMH, Trankjær H, Almarsdóttir AB, m.fl. Managing cardiovascular risk factors in patients with chronic kidney disease: pharmacological and non-pharmacological interventions in the Copenhagen CKD Cohort. Clin Kidney J. 2. juli 2024;17(7):sfae158.

5. Vestergaard SV, Christiansen CF, Thomsen RW, Birn H, Heide-Jørgensen U. Identification of Patients with CKD in Medical Databases: A Comparison of Different Algorithms. Clin J Am Soc Nephrol. april 2021;16(4):543–51.

6. Stevens PE, Ahmed SB, Carrero JJ, Foster B, Francis A, Hall RK, m.fl. KDIGO 2024 Clinical Practice Guideline for the Evaluation and Management of Chronic Kidney Disease. Kidney Int. april 2024;105(4):S117–314.

7. Illemann Christensen A. SDU. 2022 [henvist 15. januar 2025]. Vi bliver højere og tungere. Tilgængelig hos: https://www.sdu.dk/da/sif/ugens_tal/35_2022

8. Dansk Selskab for Almen Medicin [Internet]. 2019 [henvist 15. januar 2025]. Type 2-diabetes - Opfølgning og behandling. Tilgængelig hos: https://www.dsam.dk/vejledninger/type2/diagnosen

9. Jørgensen ME, Ellervik C, Ekholm O, Johansen NB, Carstensen B. Estimates of prediabetes and undiagnosed type 2 diabetes in Denmark: The end of an epidemic or a diagnostic artefact? Scand J Public Health. februar 2020;48(1):106–12.

10. Tangri N, Peach EJ, Franzén S, Barone S, Kushner PR. Patient Management and Clinical Outcomes Associated with a Recorded Diagnosis of Stage 3 Chronic Kidney Disease: The REVEAL-CKD Study. Adv Ther. juni 2023;40(6):2869–85.

11. Fraser SDS, Roderick PJ, May CR, McIntyre N, McIntyre C, Fluck RJ, m.fl. The burden of comorbidity in people with chronic kidney disease stage 3: a cohort study. BMC Nephrol. december 2015;16(1):193.

12. Ramos M, Gerlier L, Uster A, Muttram L, Steubl D, Frankel AH, m.fl. Development and validation of a chronic kidney disease progression model using patient-level simulations. Ren Fail. 31. december 2024;46(2):2406402.

13. Chronic Kidney Disease Prognosis Consortium, Matsushita K, Velde M van der, Astor BC, Woodward M, Levey AS, m.fl. Association of estimated glomerular filtration rate and albuminuria with all-cause and cardiovascular mortality in general population cohorts: a collaborative meta-analysis. The Lancet. juni 2010;375(9731):2073–81.

14. Tangri N, Grams ME, Levey AS, Coresh J, Appel LJ, Astor BC, m.fl. Multinational Assessment of Accuracy of Equations for Predicting Risk of Kidney Failure: A Meta-analysis. JAMA. 12. januar 2016;315(2):164.

15. Matsushita K, Jassal SK, Sang Y, Ballew SH, Grams ME, Surapaneni A, m.fl. Incorporating kidney disease measures into cardiovascular risk prediction: Development and validation in 9 million adults from 72 datasets. eClinicalMedicine. oktober 2020;27:100552.

16. Hippisley-Cox J, Coupland C. Development and validation of QDiabetes-2018 risk prediction algorithm to estimate future risk of type 2 diabetes: cohort study. BMJ. 20. november 2017;j5019.

17. Vidal□Petiot E, Metzger M, Faucon A, Boffa J, Haymann J, Thervet E, m.fl. Extracellular Fluid Volume Is an Independent Determinant of Uncontrolled and Resistant Hypertension in Chronic Kidney Disease: A NephroTest Cohort Study. J Am Heart Assoc. 2. oktober 2018;7(19):e010278.

18. Runesson B, Trevisan M, Iseri K, Qureshi AR, Lindholm B, Barany P, m.fl. Fractures and their sequelae in non-dialysis-dependent chronic kidney disease: the Stockholm CREAtinine Measurement project. Nephrol Dial Transplant. 1. november 2020;35(11):1908–15.

19. Moranne O, Froissart M, Rossert J, Gauci C, Boffa JJ, Haymann JP, m.fl. Timing of Onset of CKD-Related Metabolic Complications. J Am Soc Nephrol. januar 2009;20(1):164–71.

20. Levin A, Bakris GL, Molitch M, Smulders M, Tian J, Williams LA, m.fl. Prevalence of abnormal serum vitamin D, PTH, calcium, and phosphorus in patients with chronic kidney disease: Results of the study to evaluate early kidney disease. Kidney Int. januar 2007;71(1):31–8.

21. Xu H, Gasparini A, Ishigami J, Mzayen K, Su G, Barany P, m.fl. eGFR and the Risk of Community-Acquired Infections. Clin J Am Soc Nephrol. september 2017;12(9):1399–408.

22. James MT, Grams ME, Woodward M, Elley CR, Green JA, Wheeler DC, m.fl. A Meta-analysis of the Association of Estimated GFR, Albuminuria, Diabetes Mellitus, and Hypertension With Acute Kidney Injury. Am J Kidney Dis. oktober 2015;66(4):602–12.

23. Vestergaard SV, Heide-Jørgensen U, Van Haalen H, James G, Hedman K, Birn H, m.fl. Risk of Anemia in Patients with Newly Identified Chronic Kidney Disease – A Population-Based Cohort Study. Clin Epidemiol. september 2020;Volume 12:953–62.

24. Jing J, Kielstein JT, Schultheiss UT, Sitter T, Titze SI, Schaeffner ES, m.fl. Prevalence and correlates of gout in a large cohort of patients with chronic kidney disease: the German Chronic Kidney Disease (GCKD) study. Nephrol Dial Transplant. 1. april 2015;30(4):613–21.

25. Pollock C, James G, Garcia Sanchez JJ, Carrero JJ, Arnold M, Lam CSP, m.fl. Healthcare resource utilisation and related costs of patients with CKD from the UK: a report from the DISCOVER CKD retrospective cohort. Clin Kidney J. 28. oktober 2022;15(11):2124–34.

26. Sundheds- og ældreøkonomisk analyse - Kontaktmønstre på tværs af sektorer blandt befolkningen, kronikere og ældre medicinske patienter [Internet]. Sundheds- og ældreministeriet; 2018 [henvist 4. februar 2024]. Tilgængelig hos: https://www.ism.dk/Media/0/4/Sundheds-og-aeldreoekonomisk-analyse-okt-2018.pdf

27. Honorartabel (Dagtid) - Overenskomst om almen praksis [Internet]. Praktiserende Lægers Organisation; 2024 [henvist 4. februar 2025]. Tilgængelig hos: https://laeger.dk/media/hphdra0x/honorartabel-2024-april.pdf

28. Lægemiddelstyrelsen. Medicinpriser.dk [Internet]. Tilgængelig hos: https://www.medicinpriser.dk/

29. Gjeruldsen B. Peritonealdialyse – Ordination af behandling [Internet]. Region Nordjylland; [henvist 4. februar 2024]. Tilgængelig hos: https://pri.rn.dk/Sider/7003.aspx

30. DNSLs styregruppe. Dansk Nefrologisk Selskabs Landsregister (DNSL) - Landsdækkende database for patienter med kronisk nyresvigt [Internet]. RKKP; 2024. Tilgængelig hos: https://www.sundhed.dk/content/cms/92/4692_dnsl-aarsrapport-2023-offentliggjort-version-20240628.pdf

31. The Danish Health Data Authority. DRG-tariffs 2024 [Internet]. 2023 [henvist 28. februar 2024]. Tilgængelig hos: https://sundhedsdatastyrelsen.dk/da/afregning-og-finansiering/takster-drg/takster-2024

32. Ehlers LH, Lamotte M, Monteiro S, Sandgaard S, Holmgaard P, Frary EC, m.fl. The Cost-Effectiveness of Empagliflozin Versus Liraglutide Treatment in People with Type 2 Diabetes and Established Cardiovascular Disease. Diabetes Ther. maj 2021;12(5):1523–34.

33. Lindholt JS, Søgaard R. Clinical Benefit, Harm, and Cost Effectiveness of Screening Men for Peripheral Artery Disease: A Markov Model Based on the VIVA Trial. Eur J Vasc Endovasc Surg. juni 2021;61(6):971–9.

34. Witham MD, Band M, Chong H, Donnan PT, Hampson G, Hu MK, m.fl. Sodium bicarbonate to improve physical function in patients over 60 years with advanced chronic kidney disease: the BiCARB RCT. Health Technol Assess. juni 2020;24(27):1–90.

35. National Institute for Health and Care Excellence (NICE [Internet]. [henvist 21. februar 2025]. British National Formulary (BNF). Tilgængelig hos: https://www.nice.org.uk/bnf-uk-only

36. Morlock R, Chevalier P, Horne L, Nuevo J, Storgard C, Aiyer L, m.fl. Disease Control, Health Resource Use, Healthcare Costs, and Predictors in Gout Patients in the United States, the United Kingdom, Germany, and France: A Retrospective Analysis. Rheumatol Ther. juni 2016;3(1):53–75.

37. Kohli MA, Maschio M, Mould-Quevedo JF, Ashraf M, Drummond MF, Weinstein MC. The Cost-Effectiveness of Expanding Vaccination with a Cell-Based Influenza Vaccine to Low Risk Adults Aged 50 to 64 Years in the United Kingdom. Vaccines. 4. juni 2021;9(6):598.

38. National Institute for Health and Care Excellence (NICE [Internet]. 2022. TA775-Dapagliflozin for treating chronic kidney disease.

39. Humphreys I, Akbari A, Griffiths R, Graham□Woollard D, Morgan K, Noble□Jones R, m.fl. Evaluating the cost of managing patients with cellulitis in Wales, UK : A 20□year population□scale study. Int Wound J. august 2023;20(6):2129–40.

40. National Health Service [Internet]. 2022 [henvist 21. februar 2025]. NHS Costs 2020/202. Tilgængelig hos: https://www.england.nhs.uk/publication/2020-21-national-cost-collection-data-publication/

41. Roxadustat (Evrenzo) - Symptomatisk anæmi hos voksne med kronisk nyresygdom [Internet]. Medicinrådet; 2023 [henvist 21. februar 2025]. Tilgængelig hos: https://medicinraadet.dk/anbefalinger-og-vejledninger/laegemidler-og-indikationsudvidelser/r/roxadustat-evrenzo-symptomatisk-anaemi-hos-voksne-med-kronisk-nyresygdom

42. Jesky MD, Dutton M, Dasgupta I, Yadav P, Ng KP, Fenton A, m.fl. Health-Related Quality of Life Impacts Mortality but Not Progression to End-Stage Renal Disease in Pre-Dialysis Chronic Kidney Disease: A Prospective Observational Study. Shimosawa T, redaktør. PLOS ONE. 10. november 2016;11(11):e0165675.

43. Liem YS, Bosch JL, Myriam Hunink MG. Preference-Based Quality of Life of Patients on Renal Replacement Therapy: A Systematic Review and Meta-Analysis. Value Health. juli 2008;11(4):733–41.

44. Clarke P, Gray A, Holman R. Estimating Utility Values for Health States of Type 2 Diabetic Patients Using the EQ-5D (UKPDS 62). Med Decis Making. 1. juli 2002;22(4):340–9.

45. Beaudet A, Clegg J, Thuresson PO, Lloyd A, McEwan P. Review of Utility Values for Economic Modeling in Type 2 Diabetes. Value Health. juni 2014;17(4):462–70.

46. Sullivan PW, Ghushchyan VH. EQ-5D Scores for Diabetes-Related Comorbidities. Value Health. december 2016;19(8):1002–8.

47. Bagust A, Beale S. Modelling EuroQol health□related utility values for diabetic complications from CODE□2 data. Health Econ. marts 2005;14(3):217–30.

48. TA780-Nivolumab with ipilimumab for untreated advanced renal cell carcinoma 2021 [Internet]. National Institute of Health and Care Excellence (NICE); 2021 [henvist 4. marts 2025]. Tilgængelig hos: https://www.nice.org.uk/guidance/ta780/documents/committee-paper

49. Peasgood T, Brennan A, Mansell P, Elliott J, Basarir H, Kruger J. The Impact of Diabetes-Related Complications on Preference-Based Measures of Health-Related Quality of Life in Adults with Type I Diabetes. Med Decis Making. november 2016;36(8):1020–33.
